# Breed-related expression patterns of Ki67, γH2AX, and p21 during ageing in the canine liver

**DOI:** 10.1007/s11259-020-09784-x

**Published:** 2020-12-10

**Authors:** Laura J. A. Hardwick, Andre J. Kortum, Fernando Constantino-Casas, Penny J. Watson

**Affiliations:** 1 Department of Veterinary Medicine, University of Cambridge, Madingley Road, Cambridge, CB3 0ES UK; 2 Peterhouse, University of Cambridge, Trumpington Street, Cambridge, CB2 1RD UK

**Keywords:** Ageing, Canine, Ki67, Liver, Senescence

## Abstract

Cellular senescence is a molecular hallmark of ageing that is associated with multiple pathologies, and DNA damage marker γH2AX, together with cell cycle inhibitor p21, have been used as senescence markers in multiple species including dogs. Idiopathic canine chronic hepatitis has recognised breed-related differences in predisposition and prognosis, but reasons behind this are poorly understood. This retrospective study using archived post mortem tissue aimed to provide insight into liver ageing in 51 microscopically normal canine livers across seven breed categories, including those with and without increased risk of chronic hepatitis. Immunohistochemistry was conducted for γH2AX, p21, and cell proliferation marker Ki67, and the mean number of positive hepatocytes per high power field was determined. All three markers were strongly correlated to each other, but no age-dependent expression was seen in the combined study population. Overall expression levels were low in most dogs, with median values representing less than 1.5% of hepatocytes, but this increased to 20–30% in individual dogs at the upper end of the range. Individual breed differences were noted in two breeds that have increased risk of chronic hepatitis, with English Springer Spaniels having lower expression of Ki67 than other dogs, and Labradors having higher expression of Ki67 and γH2AX than other dogs. These results warrant further investigation in these breeds and highlight a need to validate reliable markers of cellular senescence in dogs.

**Supplementary Information:**

The online version contains supplementary material available at 10.1007/s11259-020-09784-x.

## Introduction

The concept of healthy ageing and longevity is an increasing focus in veterinary and human medicine (Kaeberlein et al. 2016). Chronological age is the single greatest risk factor for human disease worldwide, and clustering of spontaneous age-related chronic diseases within individuals is recognised in both humans and animals (Gilmore and Greer 2015; Kirkland et al. 2017). Among the nine interlinked molecular hallmarks of mammalian ageing is cellular senescence: a state of irreversible cell cycle arrest induced either through replicative exhaustion or through a variety of cellular stresses, but in contrast to cellular quiescence, metabolic activity is retained and a range of inflammatory mediators are released as part of the senescence-associated secretory phenotype (SASP) (Lopez-Otin et al. 2013). In the short term this serves to prevent propagation of injured cells with their subsequent clearance by the immune system, but persistence and accumulation of senescent cells is associated with multiple age-related pathologies, and as such, induced apoptosis of senescent cells in mice extends healthy lifespan (Baker et al. 2016).

A difficulty in studying cellular senescence is the lack of definitive markers, in part reflecting the complex and heterogeneous nature of senescence in different physiological settings (Munoz-Espin and Serrano 2014). The histochemical stain for senescence-associated β-galactosidase activity (SA-β-gal) has been a mainstay of *in vitro* experiments, but cannot be visualised in formalin fixed tissue that constitutes the majority of retrospective samples in veterinary research. In both human fibroblasts and mouse hepatocytes, DNA damage marker γH2AX is the best surrogate marker for SA-β-gal (Passos et al. 2007; Wang et al. 2009). Expression of γH2AX and cell cycle inhibitor p21 have also recently been used to investigate the role of senescence in the canine eye and testes (Merz et al. 2019a, b), while increased expression of p21 and γH2AX contributes to the senescence signature of *in vitro* human hepatocytes (Aravinthan et al. 2013), making these valid markers to explore.

Senescence, as defined by γH2AX expression, is reported to increase with age in mouse hepatocytes (Wang et al. 2009), and accumulation of these cells has prognostic significance in human liver diseases, with molecular mechanisms now being elucidated (Aravinthan and Alexander 2016). A previous study by our group reveals a similar increase in hepatocyte p21 expression in dogs with idiopathic chronic hepatitis (Kortum et al. 2018), and while breed-related differences in prevalence and prognosis are recognised (Bexfield et al. 2012), the aetiology, pathogenesis, and progression of this disease remains poorly understood.

We hypothesised that hepatocyte senescence may increase with age in the canine liver, and differential rates of liver ageing may give an insight into breed predisposition for idiopathic chronic hepatitis. In this retrospective study, we have characterised the expression of Ki67, γH2AX, and p21 during natural ageing in 51 microscopically normal canine livers, from seven breed categories, including those with and without a recognised increased risk of chronic hepatitis.

## Materials and methods

### Case selection

The pathology service database was searched for all dogs undergoing post mortem examination between January 2006 and October 2018, with no reported liver pathology. Dogs were recruited in seven different breed categories with the aim of spanning the widest possible size and age range. Three dogs had been included as controls in the previous hepatitis study (Kortum et al. 2018). Haematoxylin and eosin (H&E) stained sections of liver were assessed by LH under the supervision of FCC to ensure that only microscopically normal tissues were included. Samples were considered eligible if they had minimal autolysis, and no evidence of lesions including necrosis, inflammation, fibrosis, oedema, haemorrhage, hepatocyte or biliary hyperplasia, or neoplasia. Hepatic stellate cells were required to be < 10% relative to hepatocytes, and cases of macrovesicular steatosis were excluded. The age, breed, sex, and cause of death were recorded from the post mortem report.

### Histology

Sections of paraffin-embedded tissues were cut at 4 µm thickness and mounted on adhesive glass slides. Serial sections were stained using routine histological methods for H&E and rhodanine copper staining.

### Immunohistochemistry

Dewaxing, rehydration, and antigen retrieval was performed using a combined 3-in-1 Dako PT link module (Dako), and immunohistochemistry was performed manually by LH. Endogenous peroxidase activity was inhibited using EnVision FLEX Peroxidase-Blocking Reagent (Agilent Dako) for 60 minutes. Primary antibodies were incubated for one hour at room temperature: Ki67 at 1:400 (mouse monoclonal anti-Ki67, clone MIB-1, Cat no. M7240, Agilent Dako); γH2AX at 1:1000 (mouse monoclonal anti-gamma H2AX (phospho-S139) antibody, clone 9F3, Cat no. Ab26350, Abcam); and p21 at 1:100 (mouse monoclonal anti-p21, clone SX118, Cat no. M7202, Agilent Dako). For samples incubated with p21 antibody, an additional step involving 15 minutes incubation with EnVision FLEX mouse linker (Agilent Dako) was found to be optimal. An HRP-conjugated secondary antibody was applied for 30 minutes at room temperature (Dako EnVision FLEX/HRP, Agilent Dako), followed by incubation for five minutes with 3,3’-diaminobenzidine chromogen (Agilent Dako) and counterstain with Mayer’s haematoxylin for five minutes. Sections of canine skin were used as a positive control for each antibody (for example, as in (Kortum et al. 2018)), and negative control slides were prepared using commercial species-matched immunoglobulins (Agilent Dako).

### Assessment and scoring of sections

Histological scoring for rhodanine copper staining was conducted by LH and reviewed by FCC, according to (Shih et al. 2007). Hepatocyte swelling and vacuolation was noted from H&E sections and assigned a grade depending on the percentage of hepatocytes affected, with grade 1 = < 10%, grade 2 = 10–40%; grade 3 = 41–70%; grade 4 = > 70%.

**Fig. 1:**
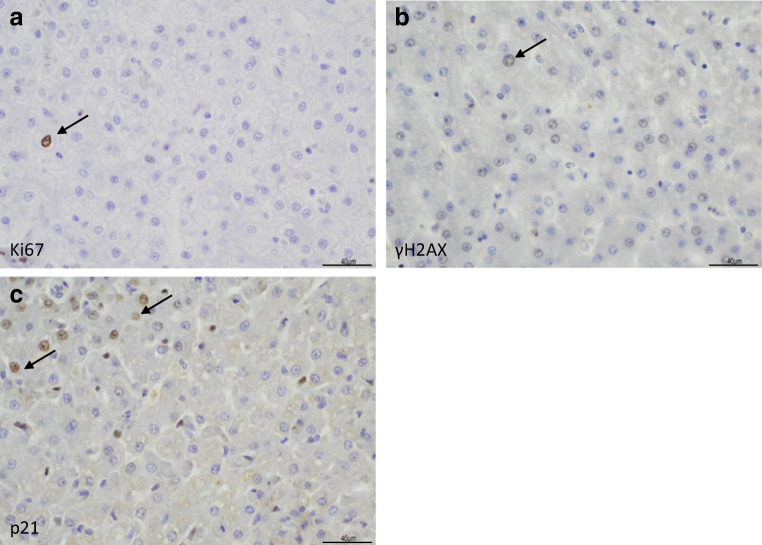
Sample high power field images showing expression of Ki67, γH2AX, and p21. Image panels illustrate expression of Ki67 (**a**), γH2AX (**b**), and p21 (**c**) with black arrows indicating cells as examples of different positive immunostaining intensities. All scale bars represent 40 µm

For immunohistochemical scoring, digital images were taken of eight random high power fields (covering 310 µm x 220 µm at 400x magnification) per marker. Scoring was conducted by LH in independent triplicate, with average scores per image recorded. LH was blinded to the breed and age of dog and each high power field (hpf) image was scored for the number of hepatocytes showing nuclear expression of the given marker. Hepatocytes were identified based on their polygonal shape, round central nucleus, and arrangement in hepatic cords. Hepatocytes with brown-stained nuclei were considered positive, irrespective of staining intensity (Fig. 1) and as conducted in (Kortum et al. 2018), but these were also subjectively assigned to either strong or weak staining intensity categories with full dataset provided in the electronic supplementary material. Using total numbers of positive hepatocytes from eight hpf, a mean number per hpf was calculated for each marker in each dog.

### Statistical analysis

Graphical illustrations and statistical analyses were performed using GraphPad Prism version 8.00 for Windows (GraphPad Software). All data were non-parametric and significance was determined using two-tailed tests, with *P* values < 0.05 considered statistically significant. Correlation between variables was assessed using Spearman correlation coefficients. Comparisons between all breed groups were performed using a Kruskal-Wallis test, or for ESS/Labradors compared to other breeds using the Mann-Whitney *U* test.

## Results

### Study population

Archived samples of microscopically normal livers from 51 dogs were included in the study (Table 1 and electronic supplementary material), with ages ranging from four months to 18 years, and representing seven different breed categories: 10 small breeds; nine English Springer Spaniels (ESS), six English Cocker Spaniels (ECS), 10 Cavalier King Charles Spaniels (CKCS), five Staffordshire Bull Terriers (SBT), seven Labradors, and four other large breed dogs. These categories were chosen to encompass small, medium, and large breed dogs, as body size influences the absolute rate of ageing, and smaller body size is associated with a longer life expectancy (Kraus et al. 2013). SBT were chosen as a breed with reduced relative risk of chronic hepatitis, while ESS, ECS, and Labradors have increased relative risk of chronic hepatitis in the UK population (Bexfield et al. 2012). Furthermore, ESS typically present in young to middle age with a severe chronic hepatitis (Bexfield et al. 2011), while Labradors generally present in middle to older age with a slower disease progression (Shih et al. 2007).

**Table 1:** Data for each of the 51 dogs with histologically normal livers

Breed	Age (years)	Mean Ki67 per hpf^a^	Mean γH2AX per hpf^a^	Mean p21 per hpf^a^
Yorkshire terrier	0.25	0.5	18.25	28.375
Yorkshire terrier	1.5	0	5	3.75
Dachshund	2.5	0	0.125	0
JRT	3	0	0.5	0.25
WHWT	6.5	0	0	0
Yorkshire terrier	7	0.125	0.25	0.375
JRT	11.5	0	0.375	0.125
Lakeland terrier	12	5.75	10	11.625
Yorkshire terrier	13.5	0.125	0.375	1.375
Yorkshire terrier	18	0	0	0
ESS	0.25	1	0.375	26.75
ESS	1.4	0	1.125	0.125
ESS	1.6	0	4.875	0
ESS	3	0	0.75	11.75
ESS	3.5	0	0	0.125
ESS	6	0	0	8.125
ESS	7.75	0	0.125	2.75
ESS	9	0	0	0.375
ESS	9.2	0	0	43.625
ECS	0.5	0	0.75	9.25
ECS	4.5	0	0.125	5.875
ECS	5	0.125	0	0.125
ECS	5.5	16.25	1.375	1.625
ECS	11	0.125	8.375	0.875
ECS	13.75	0	0	1.125
CKCS	1	0	0.25	0.625
CKCS	4	0	0	0
CKCS	3.5	0	5.75	0.25
CKCS	7.25	0	0.625	15.75
CKCS	9	0	14.875	0.625
CKCS	9.2	5.5	38.625	17.5
CKCS	11.5	0.375	0.25	8.625
CKCS	12	0.375	0.25	0
CKCS	12.25	0.125	10.5	59.25
CKCS	14	1.5	42.5	16.5
SBT	2.5	0.125	1.875	1.75
SBT	4	0	1.875	2.75
SBT	6.76	0	3.75	0.375
SBT	8.5	9.25	0	16.25
SBT	12	0.25	0	2.25
Labrador	1	1.875	35.875	38.125
Labrador	2	0.625	13	12.625
Labrador	3.5	0.5	1.125	21.125
Labrador	6	1	0.875	2.125
Labrador	10.5	7	10.125	8.5
Labrador	11	1.25	4.75	26.125
Labrador	15	0	2.875	0.125
Boxer	0.25	4	3.125	6
Boxer	3	0.25	0	0.25
German shepherd	9	1.375	0.375	6.25
Boxer	10.75	0	0.375	0.375

^a^Mean number of positive hepatocytes per hpf, scored over 8 hpf. See main text for methods

### Expression patterns of Ki67, γH2AX, and p21 in the combined study population

Immunohistochemistry was conducted using antibodies previously published in canine tissues: Ki67 as a marker of proliferating cells (Fonseca-Alves et al. 2017), γH2AX as a DNA damage marker and protein upregulated early in the senescence pathway, and p21 as a cell cycle inhibitor and protein activated downstream in the senescence cascade (Merz et al. 2019a, b).

Each high power field covered an area of 310 µm x 220 µm, and contained 150–200 hepatocytes. Ki67 staining (Fig. 1a) was observed in a random pattern of individual hepatocytes and variable staining intensity, with median 0 (range 0-16.25) positive hepatocyte per hpf. γH2AX was also seen in a random pattern, predominantly with weak staining intensity, and with median 0.625 (range 0–42) positive hepatocytes per hpf (Fig. 1b). P21 expression often showed a peri-portal distribution with variable staining intensity, and median 2.19 (range 0–59) positive hepatocytes per hpf (Fig. 1c). These median values represented less than 1.5% of hepatocytes present, but in dogs with the highest expression levels of γH2AX and p21, this represented around 20–30% of hepatocytes.

**Fig. 2:**
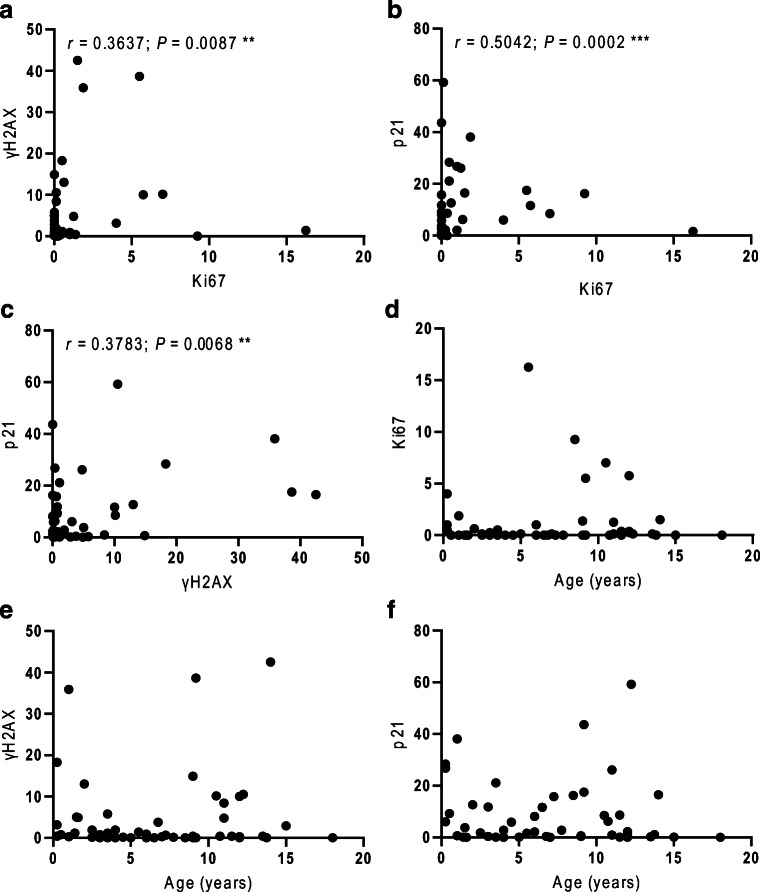
Ki67, γH2AX, and p21 were strongly correlated to each other but had no age-dependence. Positive correlations were seen between all three markers with Spearman correlation coefficient *r* and significance value *P* shown on the respective graph: (**a**) Ki67 and γH2AX, (**b**) Ki67 and p21, (**c**) γH2AX and p21. In contrast there was no correlation between age and Ki67 (**d**), γH2AX (**e**), or p21 (**f**) in the combined study population of 51 dogs

Within the combined study population of 51 dogs, Ki67, γH2AX, and p21 were strongly correlated with each other (Fig. 2a-c). Spearman correlation coefficients were as follows: Ki67 and γH2AX, *r* = 0.3637, *P* = 0.0087; Ki67 and p21, *r* = 0.5042, *P* = 0.0002; γH2AX and p21, *r* = 0.3783, *P* = 0.0068.

### Expression of Ki67, γH2AX, and p21 was not age-dependent in the combined study population

In contrast to the correlation between markers, there was no correlation between age and expression of Ki67, γH2AX, or p21 in the combined study group (Fig. 2d-f). Similarly, there was no correlation with hepatocyte vacuolation or rhodanine staining score (Electronic supplementary material), suggesting that these were not confounding factors, but we cannot exclude the possibility in this small sample size and in the absence of biochemical analysis of liver function.

### Expression of Ki67, γH2AX, and p21 was not significantly different between the seven breed groups

**Fig. 3:**
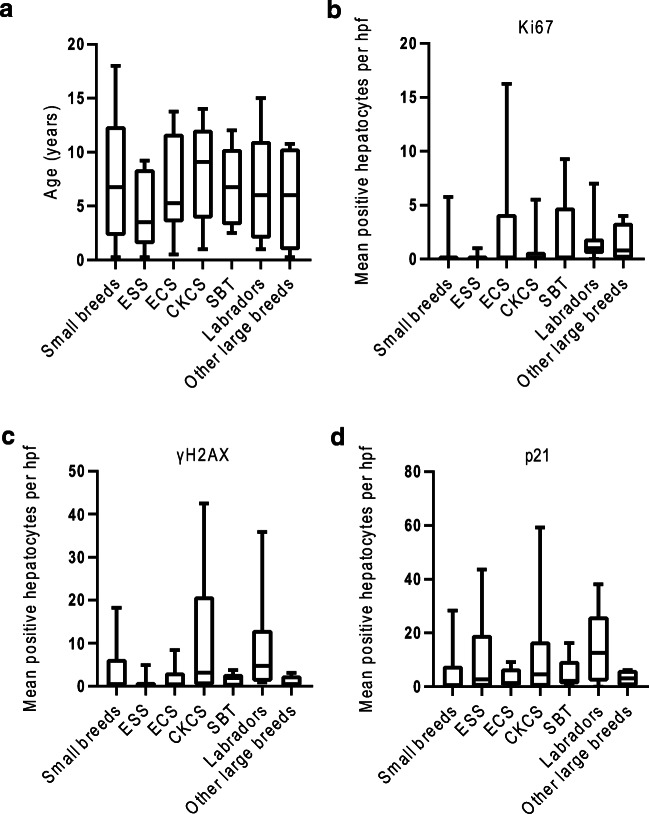
Kruskal-Wallis comparisons of all seven breed groups showed no significant differences in age, Ki67, γH2AX, or p21. Box and whiskers plots with median, interquartile range and min-max values for each breed group to compare age distribution (**a**), or expression of Ki67 (**b**), γH2AX (**c**), and p21 (**d**)

Figure 3 illustrates the data for the seven different breed categories, and Kruskal-Wallis comparison between all breed groups revealed no statistically significant differences in age, Ki67, γH2AX, or p21. Labradors had the highest median values of all three markers, indicating that breed-related trends may be present, but the small sample size within each group limited statistical power in this study.

### ESS and CKCS showed age-related changes in γH2AX and Ki67 respectively

**Fig. 4:**
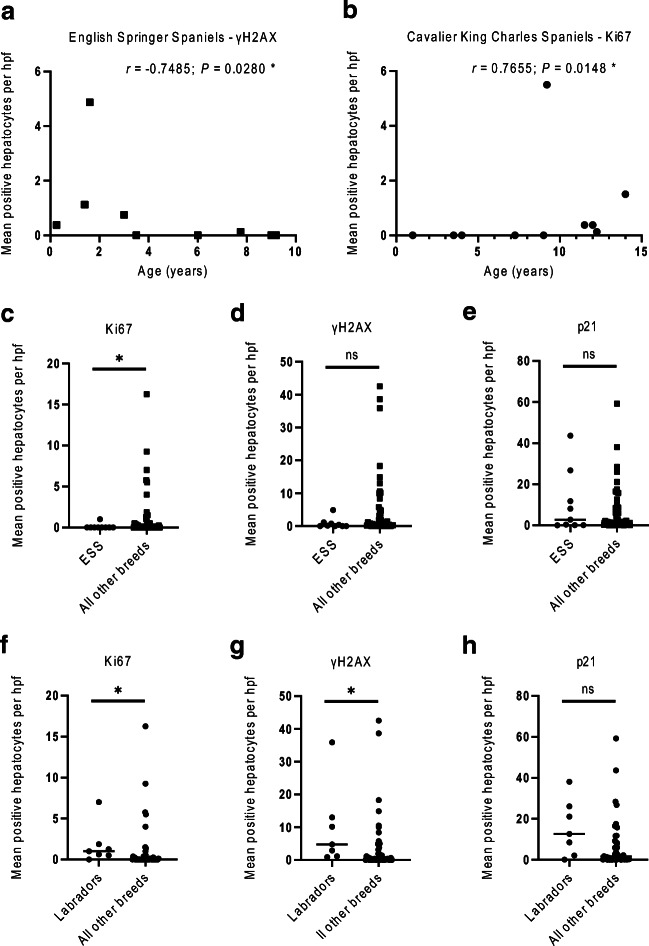
ESS, CKCS, and Labradors showed breed-specific trends in marker expression. Within breeds, ESS had a negative correlation between γH2AX and age (**a**), while CKCS had a positive correlation between Ki67 and age (**b**); Spearman correlation coefficient *r* and significance value *P* included on the respective graphs. ESS had significantly lower expression of Ki67 than all other breeds combined [*P* = 0.0218] (**c**) using the Mann-Whitney *U* test, but no significant difference in γH2AX (**d**) or p21 (**e**). Labradors had higher expression of Ki67 [*P* = 0.0132] (**f**), and γH2AX [*P* = 0.0144] (**g**), but not p21 (**h**) when compared to all other breeds using the Mann-Whitney *U* test

Age-dependence of marker expression was analysed within each breed group and identified a negative correlation between γH2AX and age in ESS (Fig. 4a; *r*=-0.7485; *P* = 0.0218), while CKCS showed a positive correlation between Ki67 and age (Fig. 4b; *r* = 0.7655; *P* = 0.0148). No other significant correlations were seen.

### ESS had lower expression of Ki67 than all other dogs

Considering individual breeds, ESS had the lowest interquartile range (IQR) and lowest maximum value for Ki67 (Fig. 3b). Data from the nine ESS dogs were compared to the other 42 dogs using the Mann-Whitney* U* test. ESS had significantly lower expression of Ki67 than all other dogs (Fig. 4c; *P* = 0.0218), and while there was a trend to reduced expression of γH2AX (Fig. 4d), this did not reach statistical significance (*P* = 0.0621).

### Labradors had higher expression of Ki67 and γH2AX than all other dogs

Similarly, Labrador-specific differences were detected when data from the seven Labradors were compared to the other 44 dogs. Labradors had higher expression of Ki67 (Fig. 4f; *P* = 0.0132) and γH2AX (Fig. 4g; *P* = 0.0144), but no significant difference in p21 compared to other dogs (Fig. 4h).

## Discussion

In this study, we have examined hepatocyte expression of Ki67, γH2AX, and p21 in microscopically normal canine livers. In the combined study population of 51 dogs, all three markers were strongly correlated with each other but not with age. Overall expression levels were low in the majority of dogs, consistent with levels reported in the normal canine testes and eye (Merz et al. 2019a, b), and individual breed differences were noted for ESS and Labradors.

We explored the expression of γH2AX and p21 as previously published indicators of cellular senescence in a range of species (Aravinthan et al. 2013; Kortum et al. 2018; Merz et al. 2019a; Wang et al. 2009), and we additionally included Ki67 as a marker of hepatocyte proliferation. Whilst most normal hepatocytes are in a state of replicative quiescence, hepatocyte turnover, and therefore Ki67 expression, may be stimulated as a result of liver injury (Delhaye et al. 1996). Scoring was conducted irrespective of staining intensity as this could be influenced by the oscillating expression with cell cycle phase in the case of Ki67 (Sobecki et al. 2017), the stage of DNA repair in the case of γH2AX (Mah et al. 2010), or simply due to differences in original sample processing given the retrospective nature of the project. There are no standardised methods of IHC scoring and we considered it important to include all labelled cells including those with weak expression, as discussed in (Fedchenko and Reifenrath 2014).

The strong correlation between expression of Ki67, γH2AX, and p21 reported here may reflect a common mechanistic link, for example in response to hepatocyte injury, with individual hepatocytes undergoing a range of cellular responses such as proliferation, or repair of DNA damage and cell cycle inhibition. Additionally, increased hepatocyte proliferation may in turn lead to replicative senescence through telomere erosion (Lopez-Otin et al. 2013), but the low number of Ki67 positive cells in this study may be more consistent with the former explanation.

We hypothesised that markers associated with cellular senescence would increase with age in the canine liver. An age-dependent increase in hepatocyte p21 expression is documented in 15 control dog livers (Kortum et al. 2018), and a similar age related increase in γH2AX-positive hepatocytes is reported in mouse liver (Wang et al. 2009). γH2AX has not previously been investigated in the canine liver, but we found no correlation between age and any of the markers in our combined study population of 51 dogs. The discrepancy with the previously published p21 study in canine liver may be due to the different study populations, both in terms of size (51 dogs versus 15 dogs) and breed composition, combined with the low overall p21 immuno-positivity in the normal canine liver. Our data indicate that senescent hepatocytes may not accumulate with age in the canine liver. A similar conclusion has recently been proposed in the canine eye (Merz et al. 2019a), and examination of hepatocyte telomere length as an indicator of replicative senescence in normal human livers reveals no age-dependent changes (Verma et al. 2012). However, our results may alternatively reflect the biological complexity of senescence in different physiological settings, and the lack of sensitive and specific markers. For example, whilst we found that Ki67, γH2AX, and p21 were strongly correlated with each other in the group as a whole, individual discordant results for γH2AX and p21 were seen in six dogs, and random versus peri-portal distribution may suggest that these two markers have independent functional significance. Dual label immuno-staining would be useful to investigate concurrent expression of γH2AX and p21 by individual cells (supportive of cellular senescence) but was unfortunately not feasible with these two primary mouse antibodies. More work is therefore needed to validate sensitive and specific senescence markers for canine tissues; an *in vitro* approach comparing candidates with telomere markers and SA-β-gal may provide further insight, but at present, care must be taken in interpretation of results.

Our second hypothesis was that breed-related differences in liver ageing may contribute to breed predispositions in idiopathic chronic hepatitis. For example, ESS typically present at a younger age and suffer a severe form of chronic hepatitis (Bexfield et al. 2011), while Labradors present in middle to older age with a more slowly progressive disease (Shih et al. 2007). ESS were found to have a lower expression of Ki67 than other dogs, and in contrast to that seen for the whole study population, γH2AX expression in ESS decreased with advancing age. One explanation for this is that as the dogs in the current study were selected to have no liver pathology, those reaching older ages may have achieved “healthy ageing”, while younger dogs may have subsequently developed liver disease had they not died of other causes. The odds ratio of ESS developing chronic hepatitis is 9.4 (Bexfield et al. 2012), and in a study of 68 cases the median age at presentation is three years seven months, ranging up to eight years five months (Bexfield et al. 2011). Five of our nine ESS were aged over three and a half with two aged over nine years. Taken as a breed irrespective of age, the lower Ki67 scores may suggest a reduced regenerative response in the ESS liver, and as such these dogs could be more prone to destructive pathology in the face of liver insults.

In contrast, increased expression of Ki67 and γH2AX was seen in the seven Labradors compared to other dogs, suggesting both increased hepatocyte proliferation and DNA damage in this breed. This may reflect behavioural factors such as increased scavenging associated with increased appetite (Raffan et al. 2016), and this may create a low grade intermittent toxic liver injury similar to alcoholic liver disease in humans. In converse to ESS, Labradors may have a robust reparative response to liver injury, and we could speculate that this may contribute to the later onset and slower progression of disease. These theories are interesting but remain unproven in this study, and it will be important for future studies to explore these possibilities with larger cohorts of dogs.

The main limitations of this study were the small sample size within each breed group, and the use of archived tissue collected from post mortem examinations. This presented potential differences in post mortem interval to sample collection, and in subsequent tissue processing. Additionally, while all samples were microscopically normal, there was a risk of sampling error with focal liver disease, and there was no biochemical information on liver function or other clinical history available for these dogs. None-the-less, this type of retrospective study provides important insights with non-invasive experimental methods.

## Conclusions

This retrospective histology-based study of natural ageing in the canine liver has revealed no age-dependent expression of Ki67, γH2AX, or p21 in the group as a whole, and overall expression levels were low. However, breed-related patterns of Ki67 and γH2AX in ESS and Labradors warrant further investigation with respect to predisposition to chronic hepatitis in these breeds. Finally, our data suggest that further work is necessary to validate reliable markers of cellular senescence in dogs.

## Electronic supplementary material

ESM 1 Electronic supplementary spreadsheet showing complete dataset categorised with strong and weak IHC staining intensity for the 51 dogs with normal livers. (XLSX 12 kb)

## Data Availability

All data generated and analysed during this study are included in this published article and its supplementary information files.
